# Demographic and Clinical Factors Associated with Reactivity of Anti-SARS-CoV-2 Antibodies in Serbian Convalescent Plasma Donors

**DOI:** 10.3390/ijerph19010042

**Published:** 2021-12-21

**Authors:** Jasmina Grujić, Nevenka Bujandrić, Zorana Budakov-Obradović, Vladimir Dolinaj, Damir Bogdan, Nebojša Savić, Alejandro Cabezas-Cruz, Dragana Mijatović, Verica Simin, Nikola Anđelić, Pavle Banović

**Affiliations:** 1 Blood Transfusion Institute Vojvodina, 21000 Novi Sad, Serbia; nevenka.bujandric@mf.uns.ac.rs (N.B.); zorana.budakov-obradovic@mf.uns.ac.rs (Z.B.-O.); 2 Faculty of Medicine in Novi Sad, University of Novi Sad, 21000 Novi Sad, Serbia; vladimir.dolinaj@mf.uns.ac.rs (V.D.); nikola.andjelic@mf.uns.ac.rs (N.A.); 3 Department of Anesthesia and Intensive Care, Clinical Centre of Vojvodina, 21000 Novi Sad, Serbia; 4 Social Sciences and Computing, University of Belgrade, 11000 Belgrade, Serbia; damirbogdan39@gmail.com; 5 Transfusion Medicine Department, Clinic for Vascular and Endovascular Surgery, Clinical Centre of Serbia, 11000 Belgrade, Serbia; nebojsabsavic@gmail.com; 6 Anses, INRAE, Ecole Nationale Vétérinaire d’Alfort, UMR BIPAR, Laboratoire de Santé Animale, F-94700 Maisons-Alfort, France; alejandro.cabezas@vet-alfort.fr; 7 Department of Prevention of Rabies and Other Infectious Diseases, Pasteur Institute Novi Sad, 21000 Novi Sad, Serbia; draganav77@gmail.com; 8 Department of Microbiology, Pasteur Institute Novi Sad, 21000 Novi Sad, Serbia; luketic.s@mts.rs; 9 Department of Microbiology with Parasitology and Immunology, Faculty of Medicine in Novi Sad, University of Novi Sad, 21000 Novi Sad, Serbia

**Keywords:** SARS-CoV-2, COVID-19, convalescent plasma, Serbia, donors, therapy

## Abstract

Passive immunotherapy with convalescent COVID-19 plasma (CCP) is used as a therapeutic procedure in many countries, including Serbia. In this study, we analyzed the association between demographic factors, COVID-19 severity and the reactivity of anti-SARS-CoV-2 antibodies (Abs) in Serbian CCP donors. Individuals (*n* = 468) recovered from confirmed SARS-CoV-2 infection, and who were willing to donate their plasma for passive immunization of COVID-19 patients were enrolled in the study. Plasma samples were tested for the presence of IgG reactive to SARS-CoV-2 spike glycoprotein (S1) and nucleocapsid antigens. Individuals were characterized according to age, gender, comorbidities, COVID-19 severity, ABO blood type and RhD factor. Total of 420 candidates (420/468; 89.74%) reached the levels of anti-SARS-CoV-2 IgG that qualified them for inclusion in CCP donation program. Further statistical analysis showed that male individuals (*p* = 0.034), older age groups (*p* < 0.001), existence of hypertension (*p* = 0.008), and severe COVID-19 (*p* = 0.000) are linked with higher levels of anti-SARS-CoV-2 Abs. These findings will guide the selection of CCP donors in Serbia. Further studies need to be conducted to assess the neutralization potency and clinical efficiency of CCP collected from Serbian donors with high anti-SARS-CoV-2 IgG reactivity.

## 1. Introduction

Severe acute respiratory syndrome coronavirus 2 (SARS-CoV-2) causing Coronavirus disease (COVID-19), appeared in Wuhan, China in late 2019 [1]. The outbreak of the disease grew very quickly into a pandemic that was officially declared by the World Health Organization (WHO) on 11 March 2020 [2]. In April 2020, the epicenter of the pandemic moved from China to the United States (US) and Europe, while the first COVID-19 death case in Europe was recorded one month earlier [3]. The severity of COVID-19 varies from asymptomatic infection to severe illness accompanied by pneumonia, hyperinflammation, hypoxemic respiratory failure, a prothrombotic state, cardiac dysfunction, substantial mortality, and persistent morbidity in some disease convalescents [4,5,6,7]. 

Due to the rapid spread of SARS-CoV-2 scientific community is given short time to develop an adequate therapeutic agent and antiviral protocols that needs to be available in every part of the world affected by pandemic. Currently there are no standardized antiviral protocols for effective COVID-19 treatment in many countries worldwide. Due to the lack of effective treatment, experimental therapeutic protocols and/or repurposed drugs are being frequently used by physicians to reduce disease severity and/or mortality. One the therapeutic protocols used for COVID-19 is passive immunotherapy using convalescent COVID-19 plasma (CCP) donated by recovered persons [8,9]. This approach is currently advised for COVID-19 patients suffering from the more severe forms of the disease in Serbia and elsewhere in the world. Due to different clinical settings and variability in anti-SARS-CoV-2 Ab levels CCP-based therapy have shown variable efficiencies in clinical trials studying the reduction in disease severity and mortality in COVID-19 patients [10,11,12,13,14]. CCP therapeutic efficiency was shown to be dependent on neutralizing anti-SARS-CoV-2 antibodies (nAbs) levels and timing of therapeutic intervention, since COVID-19 patients who received CCP with high nAbs concentration and/or from the first days after hospitalization achieved better outcomes compared to others [11,15]. 

The levels of anti-SARS-CoV-2 Abs and nAbs in the serum varies dramatically from person to person. Factors that can influence humoral immune response in SARS-CoV-2 infection are not completely understood. Several studies have shown that anti-SARS-CoV-2 Abs levels varies according to age, gender, comorbidities, disease severity, ethnicity and blood type [16,17,18,19,20,21]. The link between above mentioned parameters and reactivity of anti-SARS-CoV-2 Abs could be population dependent [10,16,22,23]. Determining the factors associated with high levels of anti-SARS-CoV-2 Abs in populations living in specific regions (e.g., Central Balkan) could assist CCP donor selection, leading to improved treatment of COVID-19 patients.

In this study we aimed at defining demographic and clinical factors associated with the reactivity of anti-SARS-CoV-2 Abs in Serbian CCP donors. 

## 2. Donors and Methods

### 2.1. Study Design

The study was conducted from 1 May 2020, to 30 April 2021, at the Blood Transfusion Institute of Vojvodina (BTIV), while partial data analysis was performed in Pasteur Institute Novi Sad, Serbia. Individuals that recovered from COVID-19 were contacted and asked if they wanted to become CCP donor candidates. CCP donor candidates underwent the same procedure as regular voluntary blood donors with additional tests that were conducted in accordance with the official Protocol approved by National Program for the CCP collection in Serbia (program name: *Doniraj plazmu #COVID19*) [24]. This includes completing a questionnaire for blood donation (Supplementary File S1), as well as medical and laboratory examination. If all findings were within referent values, person was accepted as CCP donor candidate and included for the determination of the level of Abs reactive against SARS-CoV-2 in order to determine his/her final CCP donorship status. Demographic data and data related to COVID-19 severity, blood type and comorbidities were collected during the donor preparation procedure and recorded in the BTIV information system from where they were used for analysis after receiving informed consent from each CCP candidate donor. 

### 2.2. General Criteria for Study Enrollment

Considerations to enroll individuals as CCP donor candidates were as follow. Only individuals who, (i) had a confirmed SARS-CoV-2 infection, either by real-time reverse transcriptase-polymerase chain reaction from a nasopharyngeal swab specimen or serologically detected SARS-CoV-2 IgG Abs in the serum/plasma, (ii) had a complete resolution of symptoms for at least 14 days prior to donation, (iii) were 18 years old or older, (iv) weighted 60 kg or more at the moment of the study, and (v) that successfully passed a medical examination by a physician, were enrolled in the study as CCP donor candidates. All CCP donor candidates who were recruited for the study donated their plasma for analysis within two months after recovery from COVID-19.

#### Serological and Molecular Detection of SARS-CoV-2 Infection 

For molecular detection of viral particles, SARS-CoV-2 R-GENE^®^ assay (bioMérieux, Marcy-l’Étoile, France) was used, according to previously described protocol [25]. For serological detection of anti-SARS-CoV-2 IgG Abs, SARS-CoV-2 IgG II Quant assay (Abbott, North Chicago, IL, USA) and COVID-19 ELISA IgG (Vircell S.L, Granada, Spain) assays were used, according to manufacturer instructions [26].

### 2.3. Laboratory Screening of CCP Donor Candidates

If potential CCP donor fulfilled the above-listed criteria, his/her blood sample was screened for the following parameters of interest:

#### 2.3.1. Hematological and Biochemical Parameter Screening 

Approximately 3 mL of venous blood was collected per individual in BD Vacutainer^®^ spray-coated K2EDTA tubes (Becton, Dickinson and Company, Franklin Lakes, NJ, USA). Blood cells counting was performed on Celltac Alpha MEK-6500K automated hematology analyzer (Nihon Kohden, Tokyo, Japan). For determination of coagulation status (i.e., prothrombin time, international normalized ratio, and activated partial thromboplastin time), 4.5 mL of venous blood was collected in a vacutainer tube with sodium citrate (Becton, Dickinson and Company, Franklin Lakes, NJ, USA). Coagulation status tests were conducted on fully automated hemostasis analyzer Siemens BCS XP (Siemens Healthcare GmbH, Erlangen, Germany).

About 5 mL of venous blood was collected in BD Vacutainer^®^ SST™ tubes (Becton, Dickinson and Company, Franklin Lakes, NJ, USA) and used to analyze levels of total proteins, albumin, immunoglobulins (IgG, IgM, IgA), alanine aminotransferase, aspartate aminotransferase, gamma-glutamyl transferase, total and direct bilirubin, urea, creatinine, and C-reactive protein, using spectrophotometry on Cobas Integra 400 plus fully automated analyzer (Roche Diagnostics International AG, Rotkreuz, Switzerland). 

#### 2.3.2. Screening for Markers of Transfusion-Transmitted Pathogens

Serological examinations for Human immunodeficiency virus (HIV), Hepatitis B virus (HBc), Hepatitis C virus (HCV) and Treponema pallidum exposure were performed on Alinity s analyzer using following Alinity s assays: HIV Ag/Ab Combo, Anti-HBc, Anti-HCV and Syphilis reagent kit (Abbott, North Chicago, IL, USA). Direct detection of Human immunodeficiency virus, Hepatitis B virus, Hepatitis C virus in blood samples was performed on Cobas 6800 System with Roche Cobas MPX kit (Roche Diagnostics International AG, Rotkreuz, Switzerland). All assays and appliances were used according to manufacturer instructions. 

#### 2.3.3. ABO Typing

ABO and RhD blood type were determined by performing forward and reverse complete grouping of K2EDTA-treated venous blood sample via Gel card technique using DiaClon ABO/D+ Reverse Grouping and ABD-Confirmation assay (Bio-Rad, DiaMed GmbH, Cressier, Switzerland).

#### 2.3.4. Quantification of IgG Reactive against SARS-CoV-2

Upon the first visit to BTIV, plasma of each CCP donor candidate was examined via COVID-19 ELISA IgG (Vircell S.L, Granada, Spain) to detect IgG reactive with anti-SARS-CoV-2 antigens. The used assay detected total IgG reactive with spike glycoprotein (S1 protein) and nucleocapsid (N). Before each test run serum samples were firstly inactivated at 56 °C for 30 min. One positive control, one negative control (provided by manufacturer), and duplicate cut-off control (provided by manufacturer) were included on every 96-well plate, within each test run, together with tested plasma samples. The test was considered as valid if (i) the optical density (O.D.) of the negative control was less than 0.5, (ii) the O.D. of the positive control was greater than 0.9 and both cut-off controls were greater than 0.55 and less than 1.5. Analysis result (antibody index—AI) was expressed as a ratio, calculated by dividing the O.D. of the sample by those of the mean O.D. of the cut-off controls, multiplied by 10 (as recommended by manufacturer). AI interpretation was as follows: values ≤ 6 were considered as negative, and >6 positive, as recommended by National Program for the CCP collection in Serbia.

### 2.4. COVID-19 Severity Assessment in CCP Donor Candidates

COVID-19 infection severity was classified as asymptomatic, mild illness, moderate illness, severe illness and critical illness according to the classification by US and WHO guidelines [27,28]. Data related to COVID-19 severity was accessed from central epidemiological COVID-19 database after informed consent from CCP donor candidate was obtained.

Definitions used for COVID-19 severity classification were as follows: 

Asymptomatic infection: individuals who had no symptoms that are consistent with COVID-19. 

Mild illness: symptomatic patients who have any of the various signs and symptoms of COVID-19 (fever, cough, sore throat, malaise, headache, muscle pain, nausea, vomiting, diarrhea, loss of taste and smell) without evidence of viral pneumonia or hypoxia. 

Moderate illness: individuals with clinical signs of pneumonia (fever, cough, dyspnea, shortness of breath fast breathing) who show evidence of lower respiratory disease during clinical assessment or imaging and who have an oxygen saturation (SpO2) ≥ 94% on room air at sea level. 

Severe illness: individuals who had SpO2 < 94%, a ratio of arterial partial pressure of oxygen to fraction of inspired oxygen (PaO2/FiO2) < 300 mmHg, respiratory frequency >30 breaths/min, or lung infiltrates > 50%. 

Critical illness: individuals who had respiratory failure, septic shock, and/or multiple organ dysfunction. 

### 2.5. Parameters of Interest in CCP Donor Candidates 

After CCP donor candidates were accepted for determination of anti-SARS-CoV-2 Abs, their demographic data were collected and later paired with final AI results. CCP donor candidates were grouped according to age (young adults: 18–32; adults: 33–47; and seniors: 48–60 years), gender (male and female), comorbidities (high blood pressure, diabetes, chronic obstructive pulmonary disease (COPD), and other), COVID-19 severity, ABO blood type and RhD factor affiliation.

### 2.6. Statistical Analysis

Standard descriptive values are summarized for every variable. Percentages were used for categorical variables (e.g., gender and COVID-19 severity). Continuous variables were represented via mean and 95% confidence intervals.

Individual categorical variables (age, gender, comorbidities, COVID-19 severity, ABO and RhD type) are used as independent variables in a series of t tests and ANOVAs to see if there are significant differences between the groups concerning levels of IgG reactive against SARS-CoV-2. In the case that significant difference is revealed via ANOVA test, Fisher least significant difference test (Fisher LSD) was used as a post hoc test to find means that are significantly different from each other.

The Chi-squared test was performed to determine if there is a difference between of expected and observed frequencies of the variables (age, gender, comorbidities, COVID-19 severity, ABO and RhD type) and the result of ELISA assay determining levels of IgG reactive against SARS-CoV-2 (negative/positive), while dependency within mentioned variables was assessed via contingency coefficient. The Pearson correlation coefficient was used for determination of the relationship between age of CCP donor candidates and AI value. Statistical significance was considered for *p*-values < 0.05. Data were analyzed using the SPSS software v23 (IBM, New York, NY, USA).

## 3. Results

### 3.1. Majority of Enrolled Donor Candidates Had Anti-SARS-CoV-2 Abs above Acceptance Threshold 

From a total of 468 enrolled CCP donor candidates who qualified for determination of anti-SARS-CoV-2 IgG reactivity, 420 (420/468; 89.74%) had AI values > 6 and were accepted for CCP donation. The persons who failed to reach AI values > 6 were rejected for CCP donation and therefore referred in results section as declined candidates. 

### 3.2. ABO Blood Type and RhD Factor Are Not Linked with Reactivity of Anti-SARS-CoV-2 Abs

In the whole sample of 468 candidates, blood Type A (226/468; 48.29%) and O (133/468; 28.41%) were the most frequent, while Types B (77/468; 16.45%) and AB (32/468; 6.83%) were much less frequent. The same trend was observed in the donors where the reactivity of anti-SARS-CoV-2 Abs was above the acceptance threshold (A—198/420; 47.14%, O—117/420; 27.85%, B—74/420; 17.61%, AB—31/420; 7.38%), as well as in candidates where threshold had not been reached (A—28/48; 58.33%, O—16/48; 33.33%, B—3/48; 6.25%, AB—1/48; 2.08%).

**Figure 1:**
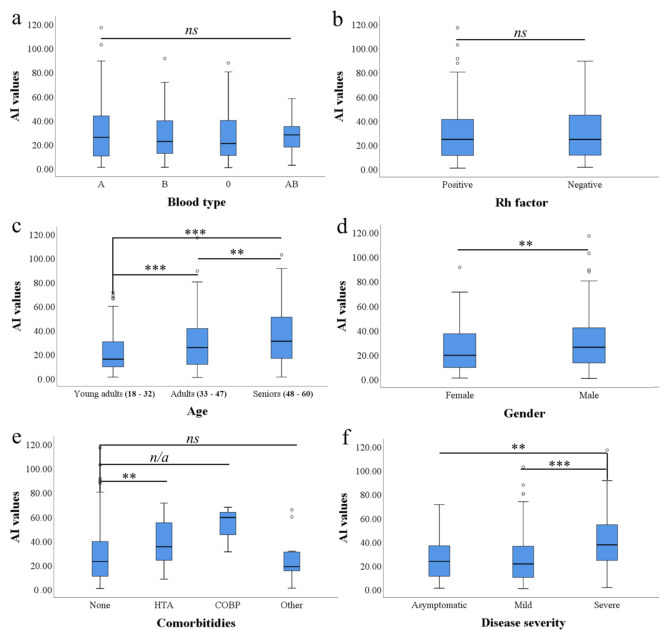
Median antibody index (AI) levels against SARS-CoV-2 comparison according to different CCP donor candidate characteristics. (**a**) Between ABO blood types; (**b**) According to Rh factor presence; (**c**) Between different age groups; (**d**) between genders; (**e**) According to the presence of comorbidity; (**f**) According to the COVID-19 severity. Presence of statistically significant difference between the groups concerning levels of IgG reactive against SARS-CoV-2 was examined by series of *t* tests (when only 2 groups were compared) and ANOVAs (when more than 2 groups were compared) followed by Fisher LSD post hoc test for individual comparison (ns—non significant; n/a—not accessible due to low sample size in COBP group ; ** *p* < 0.05; *** *p* < 0.001).

We found no difference in ABO blood type frequency in CCP donors compared to declined candidates (χ2(3) = 6.653, *p* = 0.084). In addition, we found a low association between blood group type and AI value (c = 0.119). Using one-way ANOVA, we found no significant difference in mean AI values between blood groups (F (465, 3) = 0.589, *p* = 0.622) when CCP donors and declined candidates were analyzed (Figure 1a). 

Concerning the RhD factor in total examined population recovered from COVID-19 (CCP donors + declined candidates), the majority of persons were RhD positive (413/468; 88.24%), while only 55 persons were RhD negative (55/468; 11.75%). The same RhD-positive/RhD-negative distribution pattern was noticed in persons (372/420; 88.57%) who were accepted as CCP donors (48/418; 11.42%) and in declined candidates (41/48; 85.41% and 7/48; 14.58%, respectively).

We found no difference in different RhD factor frequency in candidates who were accepted as CCP donors compared to declined candidates (χ2(1) = 0.398, *p* = 0.974) (Figure 1b). In addition, we found a low association between RhD factor affiliation and candidate acceptance outcome (c = 0.029). Using a *t*-test, we found no significant difference in quantitative AI values between RhD-positive and RhD-negative candidates (t (468) = 0.032, *p* = 0.974).

Two-way ANOVA did not show significant difference in AI values between when all blood group and RhD factor combinations (i.e., A+, A−, B+, B−, O+, O−, AB+, and AB−) were compared (F (461,7) = 0.474, *p* = 0.854).

### 3.3. Age Was Associated with Reactivity of Anti-SARS-CoV-2 IgG in CCP Donor Candidates

From a total of 468 CCP donor candidates, adults were a most frequent age group (227/468; 48.50%), followed by young adults and seniors (129/468; 27.56% and 112/468; 23.93%, respectively).

Adults also consist age group majority in candidates who achieved AI values higher than 6 (205/420: 48.80%), thus the frequency of young adults and seniors accepted as CCP donors is almost equal (109/420; 25.95% and 106/420; 25.23%, respectively). The average age of candidates who were accepted as CCP donors is 40 years (95% CI 39–41).

**Figure 2:**
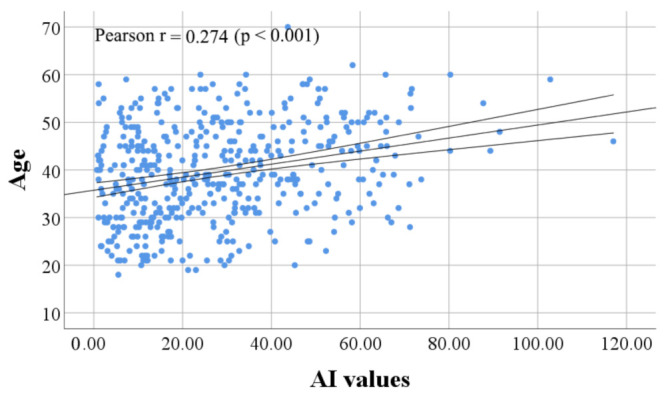
Older age is linked with higher generation of anti-SARS-CoV-2 Abs in Serbian CCP donors. Correlation analysis produce an upward slope on a scatterplot. Pearson coefficient r and *p* values are indicated.

Frequency of adult and young adult age group is almost equal in candidates who failed to achieve AI values higher than 6 (22/48; 45.83% and 20/48; 41.66%, respectively), while seniors were least frequent (6/48; 12.5%). The average age of candidates who were declined for CCP donation due to low IgG reactivity against SARS-CoV-2 was 36 years (95% CI 33.2–38.8). We found a statistically significant difference in mean age between candidate groups who failed and succeeded to reach AI threshold value (t (468) = 2.784, *p* = 0.006). Pearson correlation coefficient showed a statistically significant relationship (r = 0.274, *p* < 0.001) between the age of CCP donor candidates and the level of anti-SARS-CoV-2 Abs (Figure 2). One-way ANOVA found significant difference in mean AI values between age groups (F (466,2) = 15.570, *p* = 0.000). Fisher LSD test showed statistically significant difference in mean AI values between young adults and adults (*p* = 0.001, 95% CI 3.20–11.75), young adults and seniors (*p* = 0.000, 95% CI 9.16–19.18), as well as between adults and seniors (*p* = 0.003, 95% CI 2.21–11.17) (Figure 1c).

### 3.4. Men Show Higher Anti-SARS-CoV-2 IgG Reactivity Than Women

From a total of 468 enrolled CCP donor candidates, men were in majority compared to women (338/468; 72.22% vs. 130/468; 27.77%). A similar trend of men and women distribution was found in groups who achieved AI values above the acceptance threshold (312/420; 74.28% and 108/420; 25.71%, respectively). In contrast, men and women distribution in the group who failed to reach the required anti-SARS-CoV-2 IgG reactivity was almost equal (26/48; 54.16% and 22/48; 45.83%). A significant difference was found in men and women frequency below and above AI threshold value (χ2(1) = 8.874, *p* = 0.003). Although, the association between gender and CCP donor candidate position below/above AI threshold value is found to be low (c = 0.137).

Mean AI value was found to be higher in men compared to women (29.29; 95% CI 27.1–31.4 vs. 24.87; 95% CI 21.4–28.3, respectively) (Figure 1d). In addition, we found statistically significant difference in anti-SARS-CoV-2 reactivity between men and women (t (466) = 2.122, *p* = 0.034).

### 3.5. Comorbidities and Anti-SARS-CoV-2 IgG Reactivity 

From a total of 468 CCP donor candidate comorbidity was present in 52 cases (52/468; 11.11%). Among them, hypertension (HTA) was most prevalent (38/468; 8.11%), followed by COPD (3/468; 0.64%), while none of the CCP donor candidates had diabetes (0/468; 0%). Comorbidity within category “other” was present in 11 cases (11/468; 2.35%).

Based only on 3 samples, the highest mean AI value was detected in a group with COPD (53.76; 95% CI 35.9–71.7). Mean AI in a group with HTA and in a group without reported comorbidities was lower (37.87; 95% CI 32.2–43.5, and 27.07; 95% CI 25.1–29, respectively). The lowest reactivity of anti-SARS-CoV-2 IgG was detected in group where “other” comorbidities were reported (24.63; 95% CI 12.9–36.3) (Figure 1e).

One-way ANOVA revealed significant difference in AI values between CCP donor candidate comorbidity groups (none, HTA, COPD, “other”)—F (465, 3) = 4.992, *p* = 0.002. Post-hoc analysis (Fisher LSD) showed that only HTA group had a significantly higher mean AI value than CCP donor candidates without reported comorbidities (*p* = 0.008, 95% CI 2.06–19.54). 

### 3.6. Severity of COVID-19 Is Linked with Age and Anti-SARS-CoV-2 IgG Reactivity

From 468 CCP donor candidates, a mild form of COVID-19 was observed in the majority (356/468; 76.06%), while severe and asymptomatic cases were less frequent (89/468; 19.01% and 23/468; 4.91%, respectively). The highest anti-SARS-CoV-2 IgG reactivity was found in samples from persons who recovered from severe COVID-19 (40.077; 95% CI 35.4–44.8). Lower AI values were found in recovered from asymptomatic form (28.25; 95% CI 19.6–37), while persons with mild COVID-19 had the lowest AI scores (25.41; 95% CI 23.4–27.4) (Figure 1f).

One-way ANOVA revealed significant difference in AI values between groups with different COVID-19 severity (F (466, 2) = 18.282, *p* < 0.001). Fisher LSD showed that severe COVID-19 group had significantly higher mean AI value than donor candidates with mild (*p* = 0.000, 95% CI 8.95–20.35) and asymptomatic clinical manifestation (*p* = 0.031, 95% CI 0.85–22.78). 

When age was analyzed, we found that the average age of CCP donor candidates recovered from severe COVID-19 is 43 years (95% CI 41.1–44.9), while in the asymptomatic and mild COVID-19 group average age is 40.23 years (95% CI 38–42.4) and 39 years (95% CI 37.9–40.1), respectively.

When COVID-19 clinical manifestation of CCP donor candidates was compared according to the different age groups via one-way ANOVA we found significant difference (F (466, 2) = 5.607, *p* = 0.004). Fisher LSD showed that persons who recovered from the severe COVID-19 group are significantly older than donor candidates with mild clinical presentation (*p* = 0.003, 95% CI 1.19–7.0). 

## 4. Discussion

SARS-CoV-2 has 4 essential structural proteins: S—spike (with subunits S1 and S2), N—nucleocapsid, M—membrane, E—envelope protein. Assay performed in this study detected IgG isotype targeting abundant N protein and S1 subunit of spike glycoprotein. Although N protein is crucial for SARS-CoV-2 pathogenicity [29], only Abs reactive to spike glycoprotein are currently considered as nAbs [30]. However, Abs against SARS-CoV-2 spike glycoprotein may not be the only humoral immune response component that attenuates virus pathogenicity [31], since CCP efficiency may be dependent on the diversity of immune response components, as well as binding avidity and presence of different Ab isotypes [17]. In addition to this, detection of anti-N Abs can be used as a tool for differentiation between natural SARS-CoV-2 infection and anti- SARS-CoV-2 vaccine-associated immunity. This due to the fact that most available anti- SARS-CoV-2 vaccines (e.g., BNT162b2, rAd26-S/rAd5-S, ChAdOx1, mRNA-1273) generate an immune response only against the S glycoprotein [32]. Vircell ELISA assay that detects IgG reactive with S1 and N SARS-CoV-2 antigens is reported to be highly sensitive and with high performance compared to neutralization assay [33,34]. This ELISA assay is used for the determination of anti-SARS-CoV-2 reactivity in potential CCP donors in all Serbian transfusion medicine institutes, since there are no facilities that currently perform the SARS-CoV-2 neutralization test in Serbia up to current date. 

Since the discovery of the ABO blood type system and inheritance model related to the development of specific antigens and Abs, interest for examination of blood group susceptibility toward specific diseases emerged. The susceptibility to various diseases, such as cancer, cardiovascular diseases, infections and hematologic disorders, cognitive disorders, circulatory diseases, metabolic diseases, and malaria, has been previously linked with ABO blood groups [35]. 

ABO blood type antigens are found on red blood cells, platelets, leukocytes, plasma proteins, tissues, and cell surface enzymes, and also exist in soluble form in body secretions [36]. It is already known that blood group antigens act as receptors for various pathogens and can contribute to the facilitation of intracellular uptake and adhesion of viral particles [37]. Previously, a correlation between ABO polymorphism and susceptibility to infection with SARS-CoV-1 was examined [38]. It has been reported that anti-A Abs have a protective role in viral uptake by inhibiting angiotensin-converting enzyme-2-dependent cellular adhesion to angiotensin converting enzyme-2-expressing cells, referring that people with A or AB affiliation were at increased risk of disease [38]. These results were the basis for the further studies when COVID-19 pandemic emerged. Recent reports show that there is lower susceptibility of blood Group O and higher susceptibility of blood Type A for SARS-CoV-2 ability to cause COVID-19. In addition, the same studies reported that blood Type O subjects were significantly less represented in relation to the blood Type A, compared to their distribution in the overall population [39,40,41]. The result from the Serbian cohort is in accordance with previous results, since the majority of enrolled subjects recovered from COVID-19 analyzed here are affiliated to blood type A. Nevertheless, here we found no association of anti-SARS-CoV-2 reactivity with specific ABO blood type affiliation in enrolled subjects. Similar results were reported by Li et al. when examining COVID-19 convalescents from Wuhan, China [42]. 

On the other hand, RhD phenotypes are linked to very few diseases, such as the hemolytic disease of the newborn as a result of RhD incompatibility between mother and fetus [43]. Studies have found that RhD-positive individuals are protected against the effects of latent toxoplasmosis [44]. It is still not clear whether RhD phenotype can be linked with COVID-19 severity. However, some studies suggests that RhD-negative individuals may be less susceptible to SARS-CoV-2 infection and have decreased risk for intubation and death [45,46].

Our study found no correlation between RhD phenotype and severity of COVID-19. Similar findings were reported by Boudin et al., although they examined mainly male young population, while in our study both genders in several age groups were included [47]. Other studies discovered that RhD-negative individuals are at lower risk of SARS-CoV-2 infection, but found no correlation with the severity of COVID-19 [48,49].

In contrast, Hayes et al. showed that blood Type O had significantly lower levels of anti-N IgG compared to blood type A [50]. The other two studies reported lower levels of nAbs, anti-S and anti-RBD Abs in persons with blood type O compared to subjects with blood Types AB and B [18,51]. The different conclusions of these studies may be due to the different serological tests used in Ab detection, with different ranges of sensitivity as well as the sample size tested.

Concerning donor-specific parameters that are associated with anti-SARS-CoV-2 reactivity, we found that the age of CCP donor candidates in Serbia is in negative correlation with anti-SARS-CoV-2 IgG reactivity. Moreover, it is showed that persons who reached threshold values for CCP donorship acceptance are older than candidates who were declined (40 vs. 36 years). Similar findings concerning Ab reactivity in relation to the age group were reported for COVID-19 convalescents from Germany [52], Brazil [19], US [20,53], England [21] and Austria [54].

In addition, here we reported the link between age and COVID-19 disease severity in Serbian CCP donors. The link can be easily explained if one considers that anti-SARS-CoV-2 reactivity is often found to be superior in severe COVID-19 cases compared to cases with mild forms [19,55]. In addition, older age persons are considered as a risk group for severe COVID-19 manifestation and could be more prone to impaired innate or cellular adaptive immune response [20], such as systemic hyper inflammation [56]. A similar link was reported for COVID-19 convalescents in other countries such as England, Austria and Greece [21,54,55]. In contrast, one US based study reported the highest anti-SARS-CoV-2 Ab reactivity in blood donors aging 16/17 years [16]. A study in Mexico did not find a difference in the expression of anti-SARS-CoV-2 Abs between adult and children convalescents, neither between different COVID-19 severity groups [23]. Similarly, Körper et al. reported no correlation of age with nAb levels in German CCP donors [22]. Bartleson et al. suggested that different observations related to link between age and anti-SARS-CoV-2 Ab levels could be explained if one considers that Abs have two roles during viral infections—viral clearance and antibody-mediated enhancement of viral entry into the host cells [57]. Thus, currently there is no evidence that antibody-mediated enhancement is occurring in the case of SARS-CoV-2 infection [57]. It is also observed that aged persons are prone to develop B—lymphocytes (double-negative B cells) that are able to secrete pro-inflammatory mediators such as TNF-α, IL-6 and IL-8 and have been implicated in clinical manifestation of COVID-19 [58,59,60]. Present regional variations in humoral response to SARS-CoV-2 infection support the position that each region should determine the most preferred CCP donor characteristics taking into the account circulation of specific SARS-CoV-2 strain, population genetic diversity, population welfare status and lifestyle customs, among the others as factors that could influence the amount of exposure to SARS-CoV-2, as well as individual capacity for generation of reactive Abs [61].

Considering individual comorbidities, some studies have shown that anti-SARS-CoV-2 IgG levels show a positive correlation with obesity [62] and IgM with the presence of tumor diseases [52] due to probable impaired cellular anti-SARS-CoV-2 response. In addition, it is previously reported that comorbidities can lead to an increased level of proinflammatory cytokines which can have a stimulatory effect on humoral response [63]. Nevertheless, Körper et al. did not report the link of body weight with nAbs level in CCP donors [22]. Additionally, no correlation with anti-SARS-CoV-2 Abs level was found in the persons with chronic liver disease, chronic lung disease or diabetes [52,64]. 

When comorbidities in Serbian CCP donors were analyzed, we only found the link between anti-SARS-CoV-2 IgG reactivity and HTA. Although patients with COPD had highest AI values, no further analysis was possible due to low sample size (*n* = 3). The limitation of this study is that no additional data about arterial HTA pathophysiology and medication are available. Since HTA is identified as the most prevalent factor that can lead to more severe COVID-19 manifestation [65], it is possible that this is the mechanism is leading to higher anti-SARS-CoV-2 reactivity. Although, a further examination should be conducted to reveal what is neutralizing capacity of anti-SARS-CoV-2 Abs produced in patients with HTA, especially comparing different antihypertensive medicament groups (i.e., ACE inhibitors, calcium antagonists, diuretics, etc.). In cases of “other” comorbidities no difference was found compared to healthy CCP donors. Here we had no enrolled subjects with diabetes and we did not take account of CCP donor obesity as comorbidity. For that reason, the link of anti-SARS-CoV-2 IgG reactivity with the abovementioned parameters could not be assessed in the current study. 

Similar to previously published studies, here we also report higher anti-SARS-CoV-2 reactivity in men compared to women. The immune response differs between gender and sex hormones can play important roles. It is well known that testosterone and estrogen have their part in immune-reaction. Estrogen activates B cells function and has an impact on T cells development [66]. Regulation of interleukin-1, -10 and interferon γ is also under the influence of estrogen which implies an immune-stimulatory role [67]. Testosterone often has a suppressive effect on the immune response which explains the fact that men, especially older ones, are more described as group with a higher risk of poor prognosis of the disease [68]. For that reason, it is currently hard to explain this phenomenon observed in COVID-19 patients around the world, since no direct mechanism leading to this difference is revealed up to today. Nevertheless, this parameter could be possibly utilized as CCP donor preference criteria if higher anti-SARS-CoV-2 seroreactivity is detected in one of the genders.

## 5. Conclusions

Here we reported CCP donor characteristics associated with higher IgG reactivity against SARS-CoV-2 in Serbian population. These could help health professionals in the selection of CCP donors from Serbia and nearby countries. Nevertheless, further studies need to be conducted to assess the neutralization potency and clinical efficiency of CCP collected from donors with high anti-SARS-CoV-2 IgG reactivity. Data reported here can serve as a basis for the development of neutralization assay which will determine neutralization potency of CCP acquired from Serbian donors, using at least one currently dominantly circulating SARS-CoV-2 strain. Since SARS-CoV-2 is constantly mutating and new strains with changed epitope structures are emerging, Abs generated against one strain could be relatively ineffective against another [69,70]. With effective donor selection and CCP potency determination, we will be able to access the clinical efficiency of CCP administration in Serbian COVID-19 patients in future, taking into account the need for CCP to be rapidly administered after collection, while viral strain which caused the Ab production in CCP donor is still dominant within COVID-19 patients. 

## Supplementary Materials

The following are available online at https://www.mdpi.com/article/10.3390/ijerph19010042/s1, File S1: Blood donation questionnaire.
