# Supplementary material for: Demographic and Clinical Factors Associated with Reactivity of Anti-SARS-CoV-2 Antibodies in Serbian Convalescent Plasma Donors
Source: Int J Environ Res Public Health. 2021 Dec 21;19(1):42. doi: 10.3390/ijerph19010042 (PMC8751168; doi:10.3390/ijerph19010042)
Supplement: Supplementary file 1 [file ijerph-19-00042-s001.zip › ijerph-1492427-supplementary.pdf]

SERBIAN BLOOD TRANSFUSION SERVICE

DONOR QUESTIONNAIRE FOR BLOOD, PLATELETS AND PLASMA

FOR DONOR

Donor No.: |__|__|__|__|__|__|__|__| Date: ________________

Surname (middle name) first name: ____

Unique ID number: |__|__|__|__|__|__|__|__|__|__|__|__|__| Date of birth: ___________ Sex: M F

Address: ______________________________________ State/province: _________________________

City/town: ______________ Tel. (home): _________ Tel. (home): _________ Tel. (mobile) : _________

Company/faculty/school: _______________________________ Profession: _______________________

No. of previous donations: ___________

Signature: _________________________

FOR OFFICIAL

Donor registration and admission

Blood group: ___________

Note for medical doctor: ______________________________

____

Blood/platelet/plasma unit barcode

Official signature: _________________

Hemoglobin/hematocrit tests

Copper sulphate: ___ Normal level: ___ Low level: ___

Hemoglobinometer: ___ Read off value: ___

Blood group test

A B AB O

Tech. signature: ___________________

Medical checkup

Lungs: ______ Heart ______ Blood pressure: ____/____

Weight: __________ Height: __________

Pouch type: ___________

Note: ____

Accepted

Rejected

Rejection reason: __________________

____

MD signature: ____________________

Pouch preparation

Pouch ID No.

Tech. signature: ___________________

Venipuncture

Puncture spot		Taken blood vol.	
Left arm	|__|	405-495 ml	|__|
Right arm	|__|	< 405 ml	|__|
		> 495 ml	|__|

Donation start: ________ h ________ min

Donation end: ________ h ________ min

Premature donation interruption reason: ____

MD signature: ____________________________ Tech. signature: ___________________________

FOR DONOR

With regard to your safety and the safety of the blood transfusion treatment of the patients, please, read the questionnaire and answer each question truthfully. Your answers and other personal information are fully confidential and will be used only by the authorized transfusion institution.

1.	Have you donated blood, platelet or plasma before?	Yes	No
2.	Have you ever been rejected as blood, platelet or plasma donor?	Yes	No
3.	Do you feel healthy, rested and capable to donate blood, platelet or plasma?	Yes	No
4.	Have you had a meal before coming to donate blood, platelet or plasma?	Yes	No
5.	Do you have a dangerous profession or hobby?	Yes	No
6.	Do you take any medication regularly?	Yes	No
7.	Have you taken any medication in the last 2 or 3 days (e.g., ibuprofen, caffetin, metamizole)?	Yes	No
8.	Do you take Aspirin regularly? Have you taken it in the last 5 days?	Yes	No
9.	Have you been treated or examined in the hospital before? Are you currently under examination or on sick leave?	Yes	No
10.	Have you undergone tooth extraction in the last 7 days?	Yes	No
11.	Have you had a fever over 38°C or cold, or taken any antibiotics?	Yes	No
12.	Have you had any vaccinations or other shots in the last 12 months?	Yes	No
13.	Have you rapidly lost weight in the last 6 months?	Yes	No
14.	Have you had tick bites in the last 12 months, and have you been to see a doctor because of it?	Yes	No
15.	Have you ever been treated for epilepsy (seizures), diabetes, asthma, tuberculosis, infarct, stroke, cancer, mental disorder, or malaria?	Yes	No
16.	Do you have any chronic disease of the heart, lungs, kidneys, liver, stomach and intestines, bones and joints, nervous system, blood and blood vessels?	Yes	No
17.	Have you ever had problems with the thyroid or pituitary gland, or taken any hormone treatment?	Yes	No
18.	Do you have any skin disorders or allergies?	Yes	No
19.	Do you have prolonged bleeding after an injury or spontaneous bruising?	Yes	No
20.	**In the last 6 months**, have you:		
	a) Had surgery or received blood transfusion?	Yes	No
	b) Traveled or been living abroad?	Yes	No
	c) Had acupuncture, piercing or tattoo?	Yes	No
21.	Have you had any alcoholic beverages in the last 6 hours?	Yes	No
22.	**Certain states and behaviors:**		
	a) Are you suffering or have ever suffered from hepatitis (jaundice) type A, B or C?	Yes	No
	b) Are you living with or have had any contact with a person suffering from hepatitis (jaundice)?	Yes	No
	c) Do you think you might be at risk of getting an HIV infection?	Yes	No
	d) Have you ever done any drugs?	Yes	No
	e) Have you ever taken OTC supplements for bodybuilding (steroid supplements)?	Yes	No
	f) Have you ever received money, drugs or other payment for sexual services?	Yes	No
	g) Are you aware of all the possible ways you might have been exposed to a risk for getting infectious, blood-transmissive diseases?	Yes	No
23.	**Have you had unprotected sexual intercourse in the last 6 months:**	Yes	No
	a) With an HIV positive person?	Yes	No
	b) With hepatitis (jaundice) type B or C positive person?	Yes	No
	c) With a person who takes money, drugs or other payment for sexual services?	Yes	No
	d) With a person who has ever used needles to take drugs or steroids, or anything not prescribed by their doctor?	Yes	No
	e) With a person who might have put you at risk of getting an STI?	Yes	No
	f) Have you had anal sexual intercourse in the last 6 months?	Yes	No

FOR WOMEN

24.	Are you pregnant?	Yes	No
25.	Do you currently have menstrual bleeding?	Yes	No
26.	Have you given birth or had an abortion in the last 6 months?	Yes	No

DONOR CONSENT FOR BLOOD, PLATELET AND PLASMA

I finished the Donor questionnaire for blood, platelet and plasma, am willingly donating blood, platelet and plasma, and I hereby declare that:

I have read and understood the educational materials, and have truthfully answered all the questions above, and filled in all the information;	
I have been informed that my blood will be tested on blood-transmissible diseases;	
I have been informed that I may withdraw from the donation procedure at any time before the beginning, and during the donation process;	
I have been informed about the purpose of the blood, platelet and plasma donation;	
I am aware of the common blood donation risks and possible reactions, and the scope of blood tests;	
I have been informed about the confidentiality policy regarding provided personal information;	
I have had an opportunity to ask questions;	
I am pleased with the answers to all of my questions;	
The information I provided is truthful to the best of my knowledge;	
I confirm the credibility of the information I provided.	

THANK YOU FOR DONATING

Note:
- Quit
- Refused
- Consent withdrawn

Donor signature: ____
